# Supplementary material for: Genome-wide identification, characterization and gene expression of BES1 transcription factor family in grapevine (Vitis vinifera L.)
Source: Sci Rep. 2023 Jan 5;13:240. doi: 10.1038/s41598-022-24407-y (PMC9816167; doi:10.1038/s41598-022-24407-y)
Supplement: Supplementary file 3 — Supplementary Information. [file 41598_2022_24407_MOESM3_ESM.zip › Vvi_Atr/Vitis_vinifera.PN40024.v4.dna_sm.toplevel.fa.vs.Amborella_trichopoda.AMTR1.0.dna_sm.toplevel.fa.html/Atr-AmTr_v1.0_scaffold00168.html]

|  |  |  |  |  |  |  |  |  |  |  |  |  |  |
| --- | --- | --- | --- | --- | --- | --- | --- | --- | --- | --- | --- | --- | --- |
| Duplication depth | Reference chromosome | Collinear blocks | | | | | | | | | | | |
| 0 | Atr-ERN10155 |  |  |  |  |  |  |  |  |  |  |  |  |
| 0 | Atr-ERN10156 |  |  |  |  |  |  |  |  |  |  |  |  |
| 0 | Atr-ERN10157 |  |  |  |  |  |  |  |  |  |  |  |  |
| 0 | Atr-ERN10158 |  |  |  |  |  |  |  |  |  |  |  |  |
| 0 | Atr-ERN10159 |  |  |  |  |  |  |  |  |  |  |  |  |
| 0 | Atr-ERN10160 |  |  |  |  |  |  |  |  |  |  |  |  |
| 0 | Atr-ERN10161 |  |  |  |  |  |  |  |  |  |  |  |  |
| 0 | Atr-ERN10162 |  |  |  |  |  |  |  |  |  |  |  |  |
| 0 | Atr-ERN10163 |  |  |  |  |  |  |  |  |  |  |  |  |
| 0 | Atr-ERN10164 |  |  |  |  |  |  |  |  |  |  |  |  |
| 0 | Atr-ERN10165 |  |  |  |  |  |  |  |  |  |  |  |  |
| 0 | Atr-ERN10166 |  |  |  |  |  |  |  |  |  |  |  |  |
| 0 | Atr-ERN10167 |  |  |  |  |  |  |  |  |  |  |  |  |
| 0 | Atr-ERN10168 |  |  |  |  |  |  |  |  |  |  |  |  |
| 0 | Atr-ERN10169 |  |  |  |  |  |  |  |  |  |  |  |  |
| 0 | Atr-ERN10170 |  |  |  |  |  |  |  |  |  |  |  |  |
| 0 | Atr-ERN10171 |  |  |  |  |  |  |  |  |  |  |  |  |
| 0 | Atr-ERN10172 |  |  |  |  |  |  |  |  |  |  |  |  |
| 0 | Atr-ERN10173 |  |  |  |  |  |  |  |  |  |  |  |  |
| 0 | Atr-ERN10174 |  |  |  |  |  |  |  |  |  |  |  |  |
| 0 | Atr-ERN10175 |  |  |  |  |  |  |  |  |  |  |  |  |
| 0 | Atr-ERN10176 |  |  |  |  |  |  |  |  |  |  |  |  |
| 0 | Atr-ERN10177 |  |  |  |  |  |  |  |  |  |  |  |  |
| 0 | Atr-ERN10178 |  |  |  |  |  |  |  |  |  |  |  |  |
| 0 | Atr-ERN10179 |  |  |  |  |  |  |  |  |  |  |  |  |
| 0 | Atr-ERN10180 |  |  |  |  |  |  |  |  |  |  |  |  |
| 0 | Atr-ERN10181 |  |  |  |  |  |  |  |  |  |  |  |  |
| 0 | Atr-ERN10182 |  |  |  |  |  |  |  |  |  |  |  |  |
| 0 | Atr-ERN10183 |  |  |  |  |  |  |  |  |  |  |  |  |
| 0 | Atr-ERN10184 |  |  |  |  |  |  |  |  |  |  |  |  |
| 0 | Atr-ERN10185 |  |  |  |  |  |  |  |  |  |  |  |  |
| 0 | Atr-ERN10186 |  |  |  |  |  |  |  |  |  |  |  |  |
